# Improved patient models for simulation of clinically realistic ^68^Ga-SSTR PET and ^177^Lu-PRRT SPECT studies

**DOI:** 10.1186/s40658-026-00899-9

**Published:** 2026-05-25

**Authors:** Johan Gustafsson, Philip Kalaitzidis, Selma Curkic Kapidzic, Michael Ljungberg, Katarina Sjögreen Gleisner

**Affiliations:** 1 Medical Radiation Physics, Lund, Lund University, Lund, Sweden; 2 Radiation Physics, Department of Haematology, Oncology and Radiation Physics, Skåne University Hospital, Lund, Sweden

**Keywords:** Anthropomorphic phantoms, Monte Carlo simulations, SPECT, PET, activity quantification, SIMIND Monte Carlo

## Abstract

**Background:**

The aim was to improve the realism of patient models for Monte-Carlo based evaluation of image-based quantification of tumour volume and activity in [^68^Ga]Ga-DOTA-TOC PET and [^177^Lu]Lu-DOTA-TATE SPECT images.

**Methods:**

Three phantoms from the XCAT population were used as basis. Tumour structures were obtained from segmentation of patient ^68^Ga-PET images and positioned at relevant positions in the phantoms. Respiratory motion was modelled, and the centre-of-mass of tumours displaced according to the organ motion in the respiration. Tumour activity concentrations were sampled from a log-normal distribution derived from patient data. Intra-organ non-uniform activity concentration was modelled for spleen, liver, and intestines. The full phantom models included all these aspects. For assessment of the impact of the modelled effects, simulations were also carried out for phantoms without respiratory motion (full-without-motion) and with spherical lesions without background variability (standard models). Evaluation was focussed on the impact of the models on the errors in tumour activity concentrations estimated from SPECT and PET images with and without partial-volume correction (PVC).

**Results:**

The full models were successfully realized and were found to yield a visual appearance that more closely resembled that of patients, compared to the full-without-motion and standard models. For tumours in the volume range 0 mL to 10 mL, the mean relative errors obtained for ^177^Lu-SPECT without PVC were − 51%, -38%, and − 34% for the full models, the full-without-motion models, and the standard models, respectively. Corresponding results for ^68^Ga-PET without PVC were − 34%, -25%, and − 23%. Thus, for both PET and SPECT, the negative bias in estimated tumour activity concentrations demonstrated a clear sensitivity to the taken modelling approach.

**Conclusions:**

Respiratory motion is an important factor for modelling realistic patient images, in the context of quantitative SPECT and PET. Non-spherical tumours and background variability has a minor, but measurable impact.

## Background

Monte Carlo (MC) simulation is a versatile tool for validation and evaluation of method performance, with application to both positron emission tomography (PET) and single photon emission computed tomography (SPECT) [1–6]. Two important advantages of simulations are the complete control of input, allowing for isolation of the effect of single parameters, and the possibility of evaluating performance in complex anthropomorphic geometries [7]. Both aspects are difficult to achieve with physical phantoms. Achieving clinical realism is important to enable evaluation of image processing methods in geometries representative of patients.

A key concept for medical-image quality is that evaluation should be task specific, i.e., performed with respect to a given task, and address the range of situations that can be encountered in practice [8]. Hence, it is important that both the average properties and the variability of the data used for evaluation accurately represent the specific scenario. Variability may arise from both the imaging procedure itself, e.g., the stochastic nature of photon detection, and from the range of anatomies and biological characteristics encountered in a patient population [8]. State-of-the-art MC simulation tools, e.g., the GATE and SIMIND programs [9, 10], are competent in achieving realistic deterministic and stochastic properties with respect to the camera system, but achieving patient models with sufficient variability remains a challenge [11].

Modern anthropomorphic hybrid phantoms [12] offer flexibility in terms of changing organ sizes and are able to model breathing and cardiac motion during image acquisition. However, the phantoms are still compartmentalised with uniform activity concentration and density. This compartmentalization poses challenges for task evaluation, for example detection where a variable background acts as a confounder [13, 14]. For oncological applications it is challenging to model a distribution of tumours with sufficient realism and variability in terms of size and shape, which complicates the interpretation of evaluations of methods for quantification of tumour volume or activity-concentration [15]. Overly simplified phantom models yield simulated images with a synthetic appearance that are easily discernible from their clinical counterpart, with the resulting risk of a biased performance assessment. It is not obvious that the results obtained from a MC simulation will be useful with respect to the intended task if the model of the imaged object is inadequate, even when the model of the camera system and radiation transport is detailed and well-validated.

Enhanced patient models used for simulation studies have been addressed earlier. Stute et al. [16] incorporated pulmonary tumours from clinical PET images and introduced non-uniform tumour activity-distribution by modelling tumours with and without necrotic cores. In another study by Stute et al. [17], realistic phantom anatomy, non-uniformity in normal tissue activity-concentrations, and diverse tumour shapes were derived from clinical PET and CT images. However, these patient models did not incorporate respiratory motion. In contrast, Polycarpou et al. [18] generated patient models with substantial effort towards improving the realism of the respiratory motion by introducing aperiodic and continuous motion. Le Maitre et al. [19] introduced anatomical phantom variability to the hybrid NCAT phantom and defined activity distributions based on standardised uptake values (SUVs) from clinical PET images. The realism of the patient model was further enhanced by basing tumour models on patient data and replacing the default sinusoid respiratory cycle with patient-specific respiratory signals acquired from 4D PET/CT. Each approach had its unique focus and methodology to enhance the realism of simulations for an improved conformity between simulations and clinical acquisitions. The combination of these efforts would greatly enhance the realism of patient models seen as a whole.

Somatostatin receptor (SSTR) imaging with [^68^Ga]Ga-DOTA-TATE or -TOC for diagnostics and [^177^Lu]Lu-DOTA-TATE for therapy of neuroendocrine tumours constitute a theragnostic pair that has achieved considerable clinical and research interest [20]. Eligibility for therapy is typically based on a qualitative assessment of the ^68^Ga-PET image. The use of quantitative metrics to describe the status of the disease could potentially improve patient stratification [21]. Similarly, the therapy can be better adapted to the individual patient by the use of dosimetry, i.e., the estimation of absorbed doses to tumours and organs at risk for the individual patient [22, 23], based on peri-therapeutic serial quantitative ^177^Lu-SPECT imaging. A prerequisite for evaluation metrics to hold merit is the use of well-validated methods for deriving them. Monte Carlo simulated images are useful in this regard but have, in our experience, suffered from limitations in the realism of the patient model. Hence, there is a need for the development of workflows for generating realistic and diverse simulated PET and SPECT images.

The aim of this study was to develop a workflow to generate realistic and versatile anthropomorphic source geometries for MC simulation of PET and SPECT images that could be used for the assessment of methods for quantitative image analysis. Specific requirements were: (1) the possibility of imitating non-uniform activity concentration in organs, (2) the incorporation of a variety of tumour shapes and sizes, (3) the use of realistic lesion loci with respect to the studied pathology, (4) the definition of lesion-specific activity concentration, and (5) the incorporation of respiratory motion. The primary application considered was [^68^Ga]Ga-DOTA-TOC PET and [^177^Lu]Lu-DOTA-TATE SPECT imaging, but the developed methods should be generalizable to other radiopharmaceuticals and theragnostic pairs as well.

## Methods

### Phantom design

**Fig. 1:**
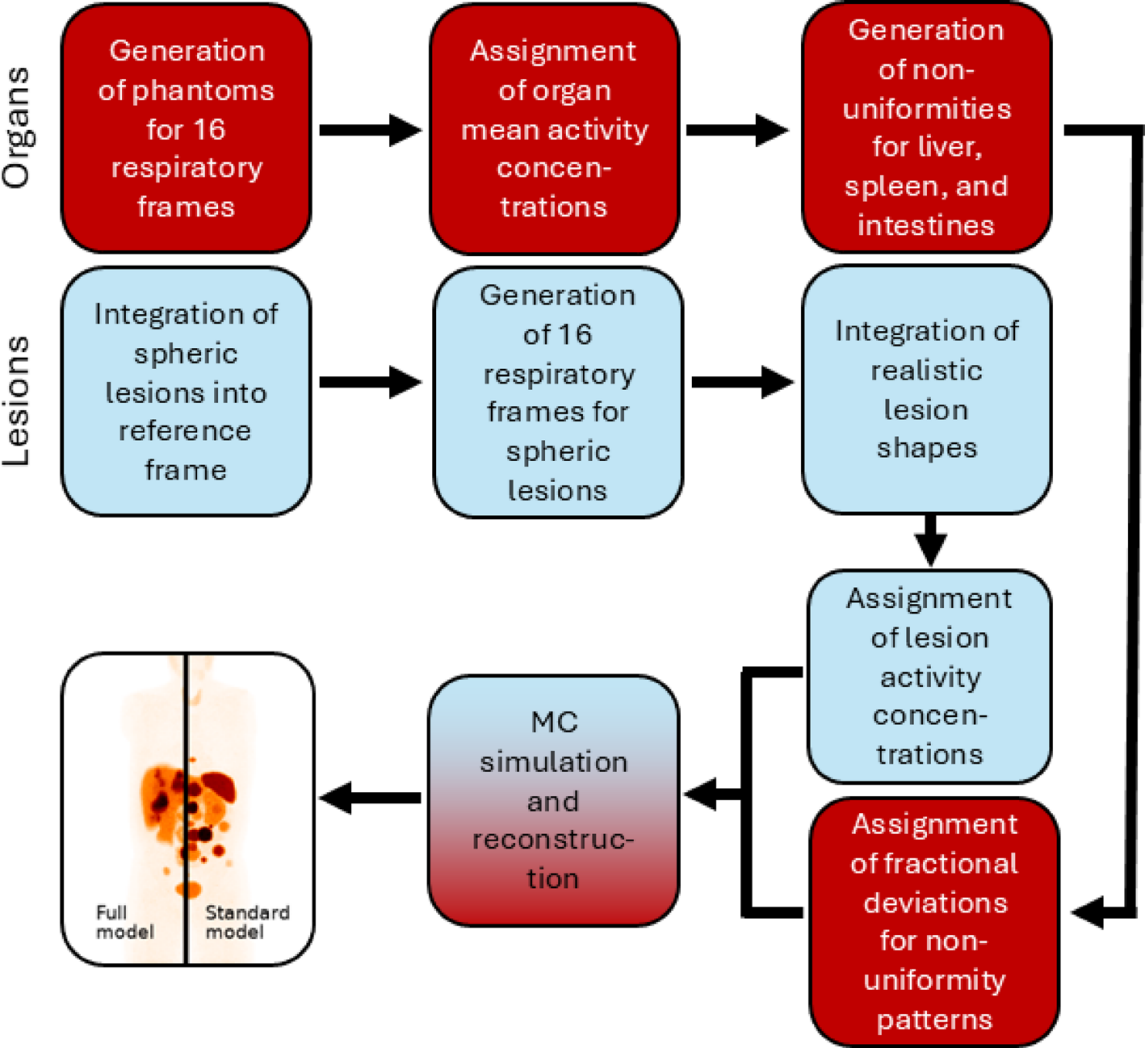
Flowchart outlining the steps for phantom generation and image simulation

The phantom generation was implemented as a multi-step process. The first step was the realization of phantoms from the XCAT population [24] and constructing a library of voxelised lesions from patients. The following steps (Fig. 1) comprised the introduction of respiratory motion, integration of lesion geometries into the breathing phantoms, assigning lesion-specific activity concentrations, and generating non-uniform intra-organ activity concentrations for selected organs.

#### Phantom generation

**Fig. 2:**
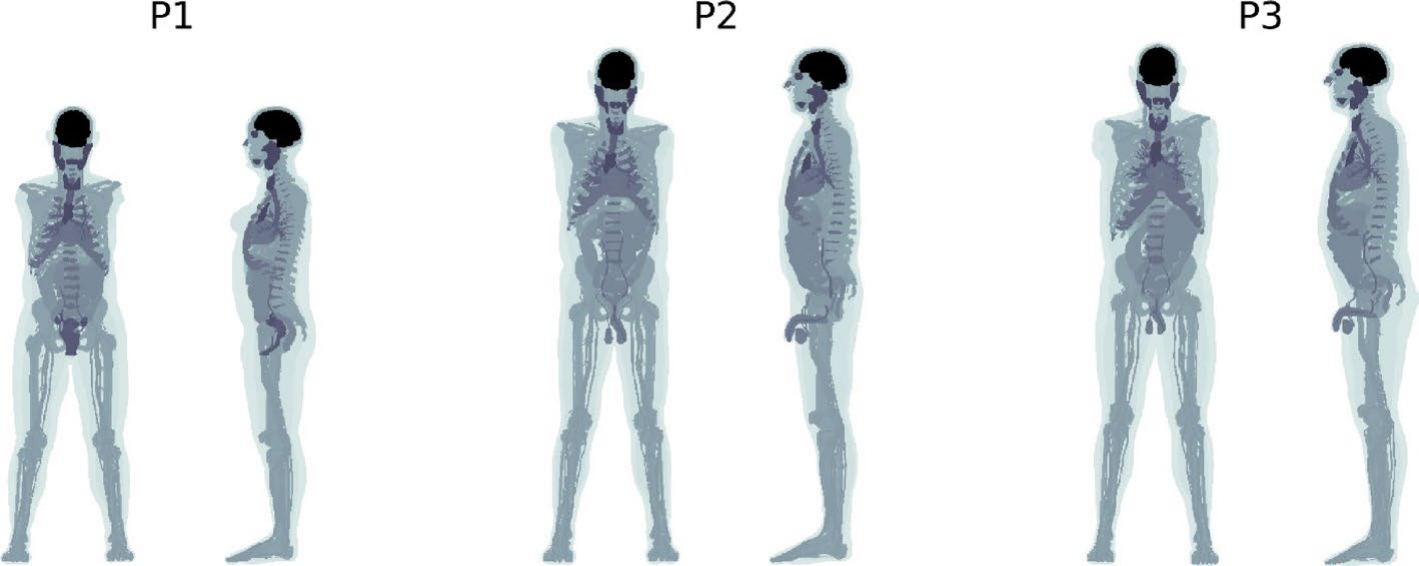
Coronal and sagittal MIPs of the code-based phantoms P1 (left), P2 (middle), and P3 (right) for a single respiratory frame

Three 4D anthropomorphic computer phantoms from the XCAT population [12, 24], henceforth referred to as P1, P2, and P3, were generated in matrices with a voxel size of 2.73 × 2.73 × 2.79 mm^3^. Respiration [12] was modelled with a complete respiratory cycle lasting 5 s divided into 16 frames using the standard respiratory-motion curves in the XCAT distribution with a maximum diaphragma displacement of 2.0 cm and a maximum chest anterior-posterior expansion of 1.2 cm. For each respiratory frame, a matrix was generated with the phantom organs displaced based on the motion of the lungs. Each phantom structure was represented with a unique code for assigning activity concentrations and densities. Coronal and sagittal maximum intensity projections (MIPs) for one respiratory frame of P1, P2, and P3 are shown in Fig. 2, and the physical characteristics of the phantoms are listed in Table 1.

**Table 1:** Physical characteristics of the phantoms

	Sex	Height/m	Weight/kg	BMI/(kg/m^2^)
P1	Female	1.59	73	29
P2	Male	1.80	90	28
P3	Male	1.82	98	30

#### Lesions

Lesion masks were obtained from delineation of 73 tumours in ^68^Ga-SSTR PET patient images. Delineation was performed using an active-surface model based on Fourier surfaces [25] as part of previous studies [26, 27]. The lesion masks were for this study realized in the same voxel size as the anthropomorphic phantoms.

Positioning of the lesions was performed by side-by-side inspection of clinical ^68^Ga-SSTR PET images and the reference frame of each phantom, with the ambition to reproduce tumour positions in patients, whilst ensuring that the lesion geometries fitted within the respective hosting organ and that there was no overlap between lesions. An in-house computer program was constructed for this purpose. Eight lesions were positioned within phantom P1, 22 lesions in P2, and 46 lesions in P3. The corresponding total lesion volumes were 163 mL, 399 mL, and 913 mL, respectively.

The XCAT program allows for the insertion of spherical lesions at arbitrary coordinates that follow the respiratory motion of the anatomy. This capability was utilised by first defining small spherical lesions and determining their centre-of-mass during the respiratory motion through the 16 frames. The patient-based lesion masks from the ^68^Ga-SSTR PET images were then integrated by matching their respective centre-of-mass with that of the corresponding sphere. Lesions positioned in skeletal structures were kept static throughout the respiratory cycle.

Mean lesion activity concentrations were assigned according to the standard uptake value (SUV) for hepatic metastases [28] and the pharmacokinetic model by Brolin et al. [29], for [^68^Ga]Ga-DOTA-TOC and [^177^Lu]Lu-DOTA-TATE, respectively. Variability between lesions was achieved by random sampling of relative deviations from a log-normal distribution with a mean of unity and a standard deviation of 0.624, as derived from the set of tumour activity concentrations per injected activity reported in Stenvall et al. [27].

#### Organs

Organ mean activity concentrations for [^68^Ga]Ga-DOTA-TOC were assigned following Kalaitzidis et al. [30], with imaging performed 60 min post-administration. Normal-organ mean activity concentrations for [^177^Lu]Lu-DOTA-TATE were based on activity concentrations from Brolin et al. [29].

Intra-organ non-uniform activity concentration was introduced for liver, spleen, and intestines. Three-dimensional randomly varying patterns, $$ F(x, y, z)$$, were created similarly as in Leube et al. [31] using Fourier series according to

1 $$\begin{array}{*{20}c} {F\left( {x,y,z} \right) = \mathop \sum \limits_{{i = - M}}^{M} \mathop \sum \limits_{{j = - M}}^{M} \mathop \sum \limits_{{k = - M}}^{M} g_{{i,j,k}} \frac{{\cos \left( {2\pi \frac{{ix + jy + kz}}{p} + \varphi _{{i,j,k}} } \right)}}{{\left( {i^{2} + j^{2} + k^{2} } \right)^{\beta } }}} \\ \end{array}, $$

where *g*_*i*,* j*,*k*_ is a random Gaussian-distributed number with mean 0 and standard deviation 1, *φ*_*i*,* j*,*k*_ is a random phase shift between -*π* and *π*, *p* is the period of the Fourier series, and *x*,* y*, and *z* represent the voxel index. *M* and *β* are adjustable parameters governing the cut-off spatial frequency and the rate of attenuation for high frequencies, respectively.

The 3D patterns were constructed with the same dimensions as the phantoms and confined to the respective organ by multiplication with its binary mask. The mean activity concentration was adjusted linearly to achieve a continuous variation within the respective organ. This method of introducing physiological variation was performed for the liver and spleen.

Intestinal uptake for [^68^Ga]Ga-DOTA-TOC and [^177^Lu]Lu-DOTA-TATE is typically patchy [32]. To mimic this pattern, a threshold was introduced such that the intestinal activity was assigned only to voxels above the threshold in the random 3D pattern. The threshold was adjusted such that 20% of the intestinal volume was assigned activity while 80% was assigned an activity concentration of zero. Table 2 presents the parameters used with Eq. (1) for each specific organ and the maximum permitted deviation from the mean activity concentration (Δ).

**Table 2:** Parameters used to create the randomised pattern by Eq. (1) for the liver, spleen, and intestines

	Liver	Spleen	Intestines
M	16	16	4
$\beta$	0.4	0.4	0.4
*p*	256	256	256
${\Delta }$	20%	10%	NA

### Monte Carlo simulations

The PET computational chain from simulation to reconstructed images has been described previously [33]. PET simulations were performed for a model of a GE Discovery MI PET/CT (GE Healthcare) with four rings using the Gate program [9]. Each phantom was imaged over either 5 (P1) or 6 (P2 and P3) bed positions with a 46 mm overlap and an acquisition of 3 min per bed position, from mid-thigh to the head.

SPECT simulations were performed using the SIMIND MC program [10] for a model of a GE Discovery NM/CT 670 SPECT system with 5/8” NaI(Tl) crystals with 8.6% energy resolution. The SIMIND program has been previously utilized and evaluated for ^177^Lu-SPECT simulations in, e.g., Gustafsson et al. [34], Leube et al. [35], and Ramonaheng et al. [36]. Projections were simulated for 60 angles over 360° following the patient contour with 45 s per projection and 128 × 128 matrices with a pixel size of 4.42 × 4.42 mm^2^. The energy window was centred around 208 keV with a width of ±7.5%. A medium-energy general-purpose collimator was simulated, also incorporating collimator penetration and scattering. Each phantom was simulated for a single-bed position but with the virtual camera axially extended to emulate a two-bed image acquisition covering the lower abdomen to the upper thorax.

Additional simulations of a NEMA IEC Body phantom with spherical inserts (0.52 mL to 26.52 mL) in a non-radioactive background were performed for determination of volume-dependent recovery coefficients. Sphere activity concentrations were 50 kBq mL^−1^ for PET and 19.5 MBq mL^−1^ for SPECT. Spherical volumes of interest (VOIs) with dimensions corresponding to the physical sphere dimensions were defined in the reconstructed PET and SPECT images, and the activity-concentration recovery determined for the different spheres.

### Tomographic reconstruction

PET images were reconstructed with the CASToR software v3.1 [37] using the ordered-subset expectation-maximisation (OS-EM) algorithm (3 iterations, 16 subsets), including time-of-flight information, normalization, and compensation for, randoms, scatter, and attenuation. The compensation methods and their integration with CASToR are described in detail in Kalaitzidis et al. [33]. The fourth respiratory frame from the XCAT phantoms was used to compute attenuation correction factors. The PET images were post-filtered using a Gaussian kernel with 5.5 mm transaxial full width at half maximum (FWHM) and 4 mm axial FWHM. SPECT-image reconstruction was performed using OS-EM (8 iterations, 10 subsets) with compensation for attenuation, scatter using the ESSE method, and non-perfect distance-dependent spatial resolution [38]. PET images were scaled to activity concentration by simulation and reconstruction of a uniform cylinder filled with ^18^F [30, 33], whilst the SPECT images were calibrated through simulation of a thin cylindrical layer of ^177^Lu (10 cm in diameter).

### Evaluation

Evaluation was focussed on the impact of the different model improvements for the capability to quantify the activity concentration in tumours. For this purpose, two additional models were generated by successive removal of the aspects considered in the full model (Table 3). The model denoted ‘full’ included all the steps described above, i.e., respiratory motion, patient-based lesion masks and non-uniform activity concentrations within organs and between lesions. The model denoted ‘full-without-motion‘ excluded respiratory motion and was otherwise kept as for the full model. The model termed ‘standard’ was based on spherical lesion geometries and uniform activity distributions within organs and across lesions. For the models full-without-motion and standard, the individual lesion volumes and the mean activity concentrations in organs and across lesions were kept identical to those of the full model, for the respective phantom.

Simulation of the three phantoms with the three models together yielded nine ^68^Ga-PET images and nine ^177^Lu-SPECT images. For each image, the lesion activity concentrations were estimated in VOIs derived from the respective set of lesion masks. Lesion masks were identical to those defined at simulation-input, transformed to the image coordinate system using the volume-preserving technique described in Gustafsson et al. [34]. In brief, this technique gradually includes voxels in the VOI mask according to their fractional overlap with the region defined in the simulations until the volume specified in the simulation is reached. The activity concentration was estimated both with and without post-reconstruction partial-volume correction (PVC). Partial-volume correction was performed using recovery coefficients derived from simulated NEMA phantom and using spherical VOIs with the same size as the simulated spheres to evaluate the activity concentration. Recovery was parametrized as function of volume according to

2 $$ \begin{array}{*{20}c} {RC\left( V \right) = \frac{1}{{1 + \left( {\frac{\alpha }{V}} \right)^{\beta } }},} \\ \end{array} $$

where *V* was the VOI volume and $\alpha$ and $\beta$ two parameters determined using non-linear least-squares fitting. Spill-in from background activity was accounted for assuming a linear translation-invariant system according to [39]

3 $${C_{{{\mathrm{PVC}}}} = \frac{{C_{{{\mathrm{img}}}} - C_{{{\mathrm{bkg}}}} \left( {1 - {\mathrm{RC}}} \right)}}{{{\mathrm{RC}}}}}, $$

where $C_{{{\mathrm{img}}}}$ is the lesion activity-concentration estimated directly from the image and $C_{{{\mathrm{bkg}}}}$ is the estimated background activity-concentration. The background activity-concentration was estimated as the mean concentration in a shell surrounding the lesion, created by successive dilations of the original VOI. The first dilation was performed with a spherical mask with two voxels radius and the second dilation was performed with a spherical mask with three voxels radius. The background VOI was defined as the logical difference between the two.

**Table 3:** Aspects considered for the three evaluated models

Model notation	Motion	Lesion shape	Activity distribution	
			Intra-organ	Inter-lesion
Full	Respiratory	Patient-based	Non-uniform	Non-uniform
Full-without-motion	None	Patient-based	Non-uniform	Non-uniform
Standard	None	Spherical	Uniform	Uniform

## Results

### Phantom generation

**Fig. 3:**
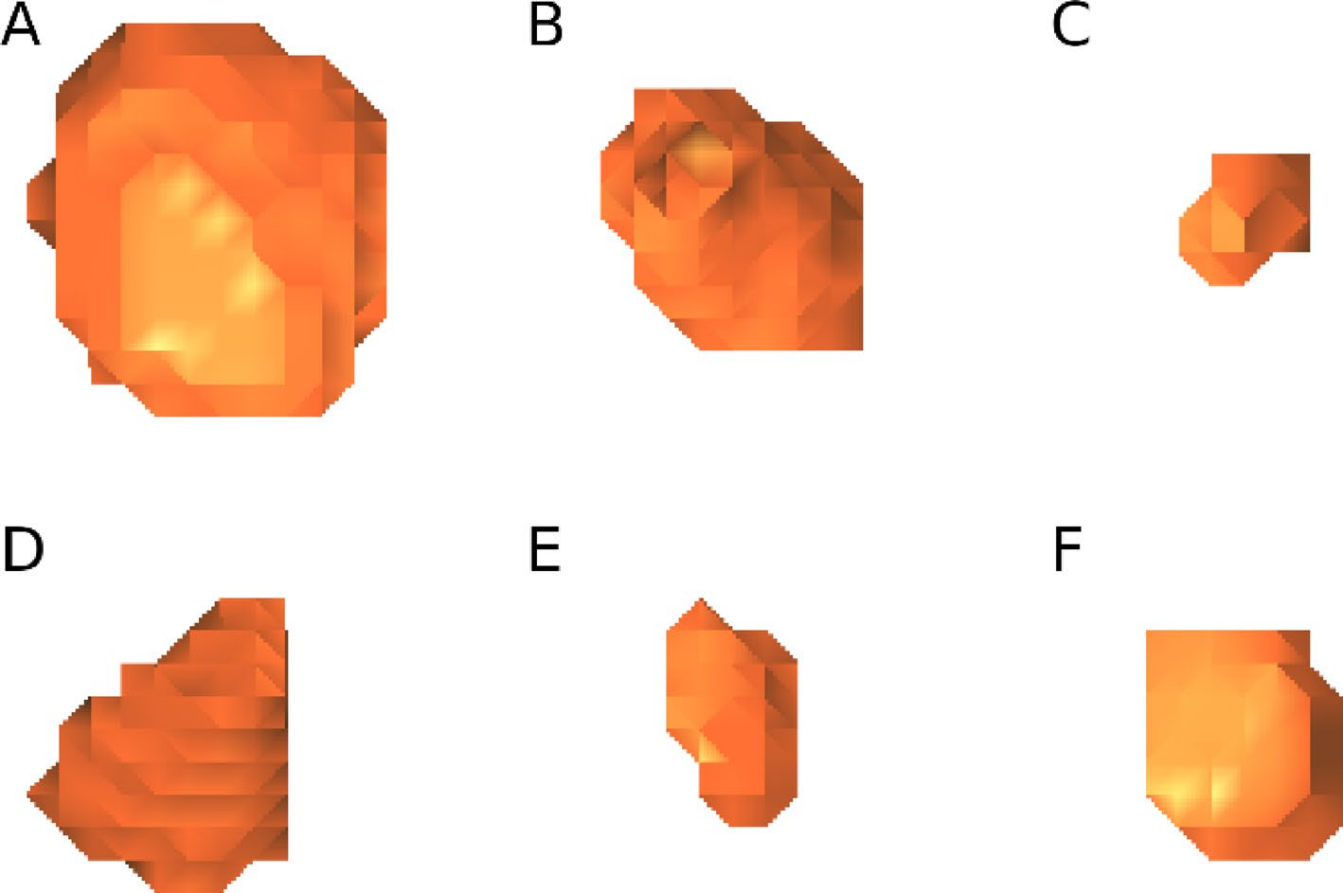
Rendering of example lesions A to F, representing various shapes and with volumes between 2.4 mL and 22 mL

**Fig. 4:**
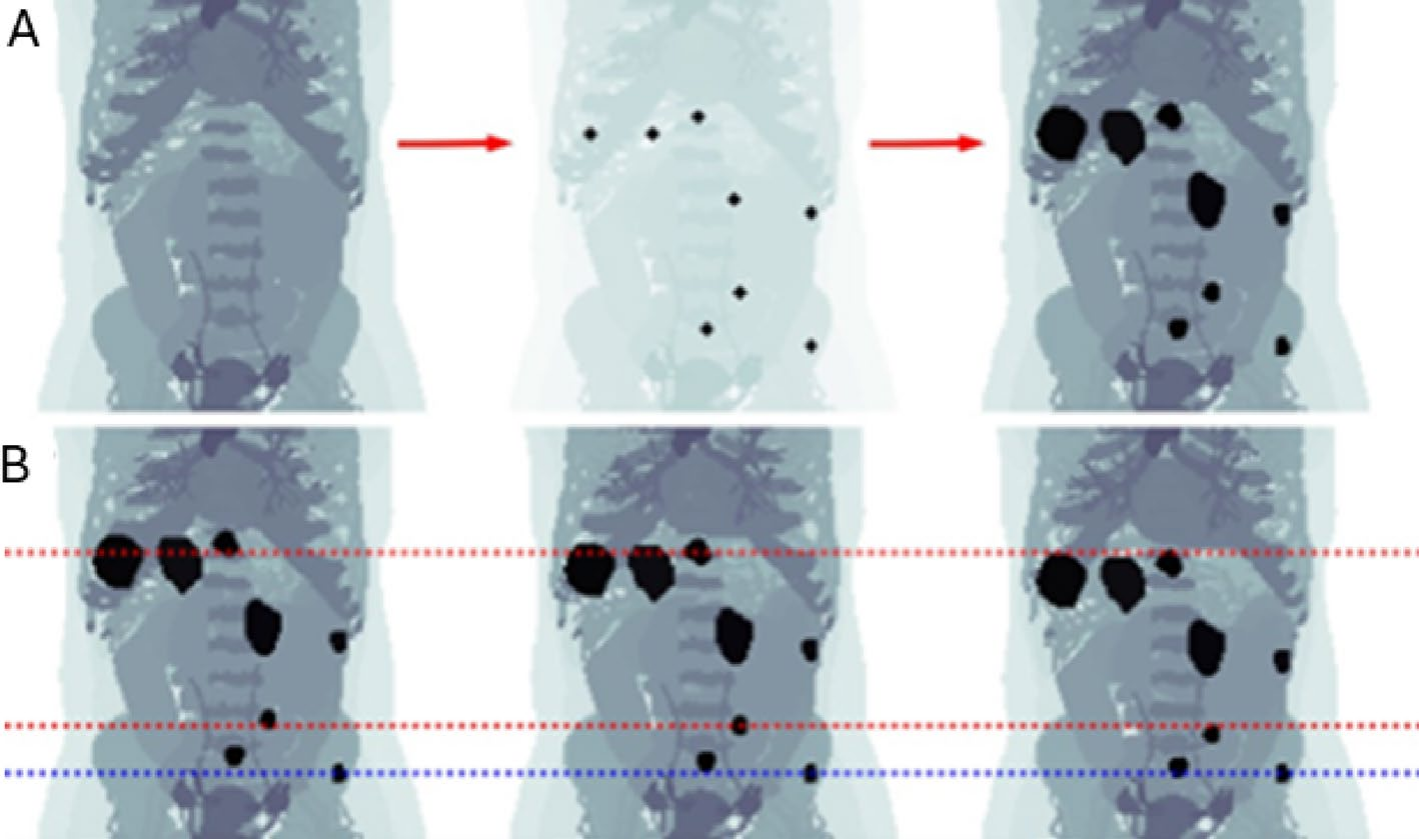
Illustration of the process for integration of realistically shaped lesions into the reference frame of phantom P1 (top row), and three respiratory frames inducing lesion motion (bottom row). Top row (**A**): Coordinates are identified by side-by-side comparison of phantoms and patient images, and respiratory motion is introduced. The centre-of-mass of the lesions are co-aligned with these coordinates, for each respiratory frame. Bottom row (**B**): Respiratory frames 1 (complete exhalation), 4, and 8 (maximum inhalation), with red lines indicating two lesions following the respiratory motion and blue line indicating a lesion in the hip bone unaffected by respiratory motion

Examples of lesion masks resulting from segmentation of patient PET images are shown in Fig. 3, representing a range of shapes and volumes. Across all tumours, volumes ranged from 0.9 mL to 171.0 mL. Figure 4 illustrates the process of lesion positioning in the phantoms with respiratory motion, starting with spherical lesions and then replacing them with realistic lesion masks.

In accordance with patient data, lesions were mainly positioned in the liver and abdomen of the phantoms. The random sampling of the patient-based log-normal distributions yielded activity concentrations ranging from − 85% to 243% of the respective mean concentrations for ^68^Ga-PET and ^177^Lu-SPECT. Tumour activity concentrations for PET simulations had a median [min, max] of 28 kBq mL^−1^ [5.3 kBq mL^−1^, 112 kBq mL^−1^] surrounded by background activity concentrations of 4.9 kBq mL^−1^ [0.042 kBq mL^−1^, 19 kBq mL^−1^]. Corresponding results for SPECT simulations were 1600 kBq mL^−1^ [280 kBq mL^−1^, 6600 kBq mL^−1^] and 64 kBq mL^−1^ [0.48 kBq mL^−1^, 380 kBq mL^−1^].

**Fig. 5:**
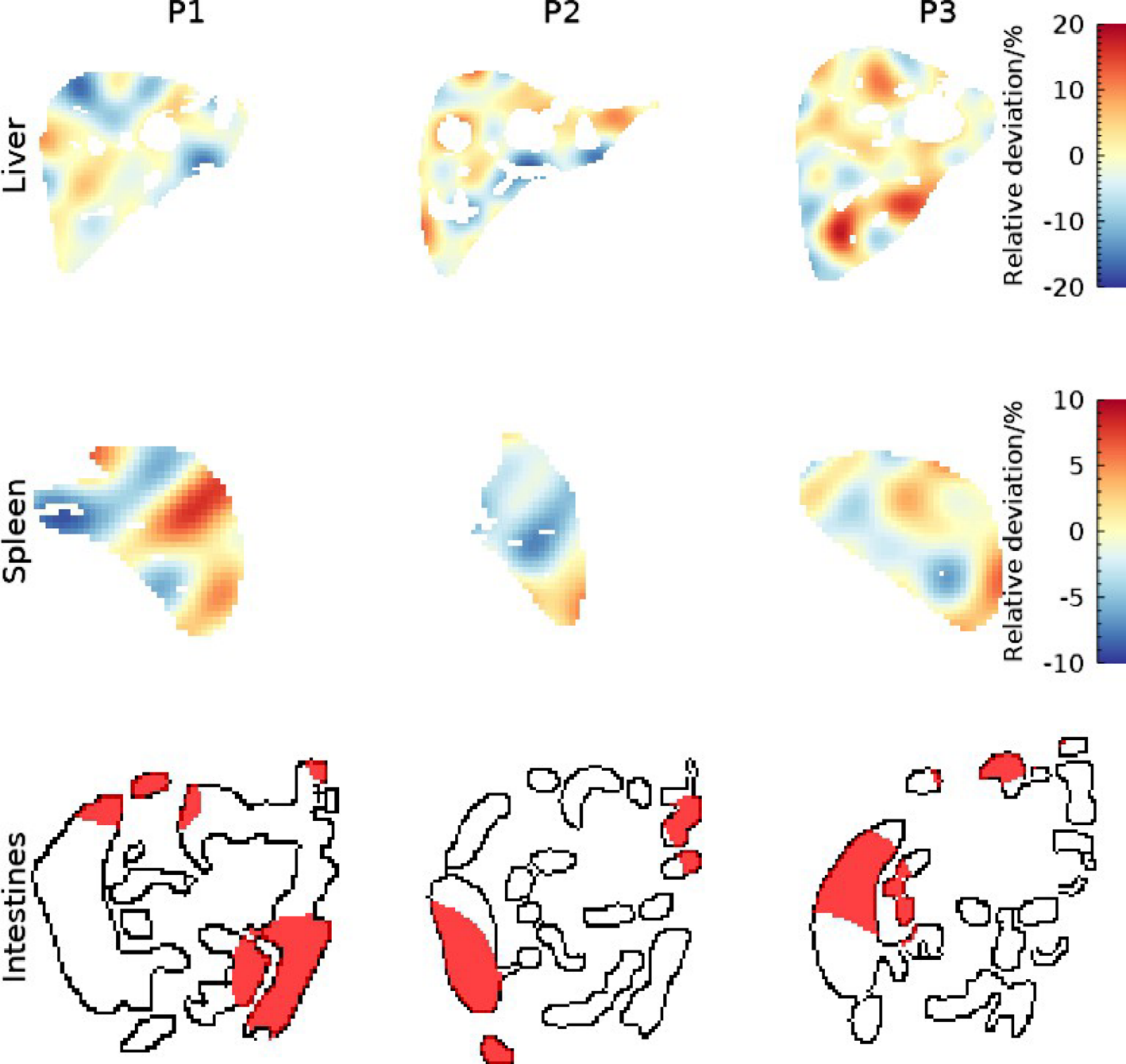
Non-uniform activity distributions for liver, spleen, and intestines for coronal slices of P1, P2, and P3. The white regions in liver and spleen correspond to phantom coordinates assigned to lesions and blood vessels, thus not included in the respective organ compartment. For intestines, black lines show the outline whilst the red regions indicate sections where the activity was located

The organ activity concentrations resulting from non-uniformity modelling for liver, spleen, and intestines are illustrated in Fig. 5. For liver and spleen, the model (Eq. 1) generated a continuously varying activity concentration, imitating an underlying biological variation in radiopharmaceutical uptake. For intestines, the introduced threshold generated a patchy pattern, which is often observed for patients.

### PET and SPECT simulation

**Fig. 6:**
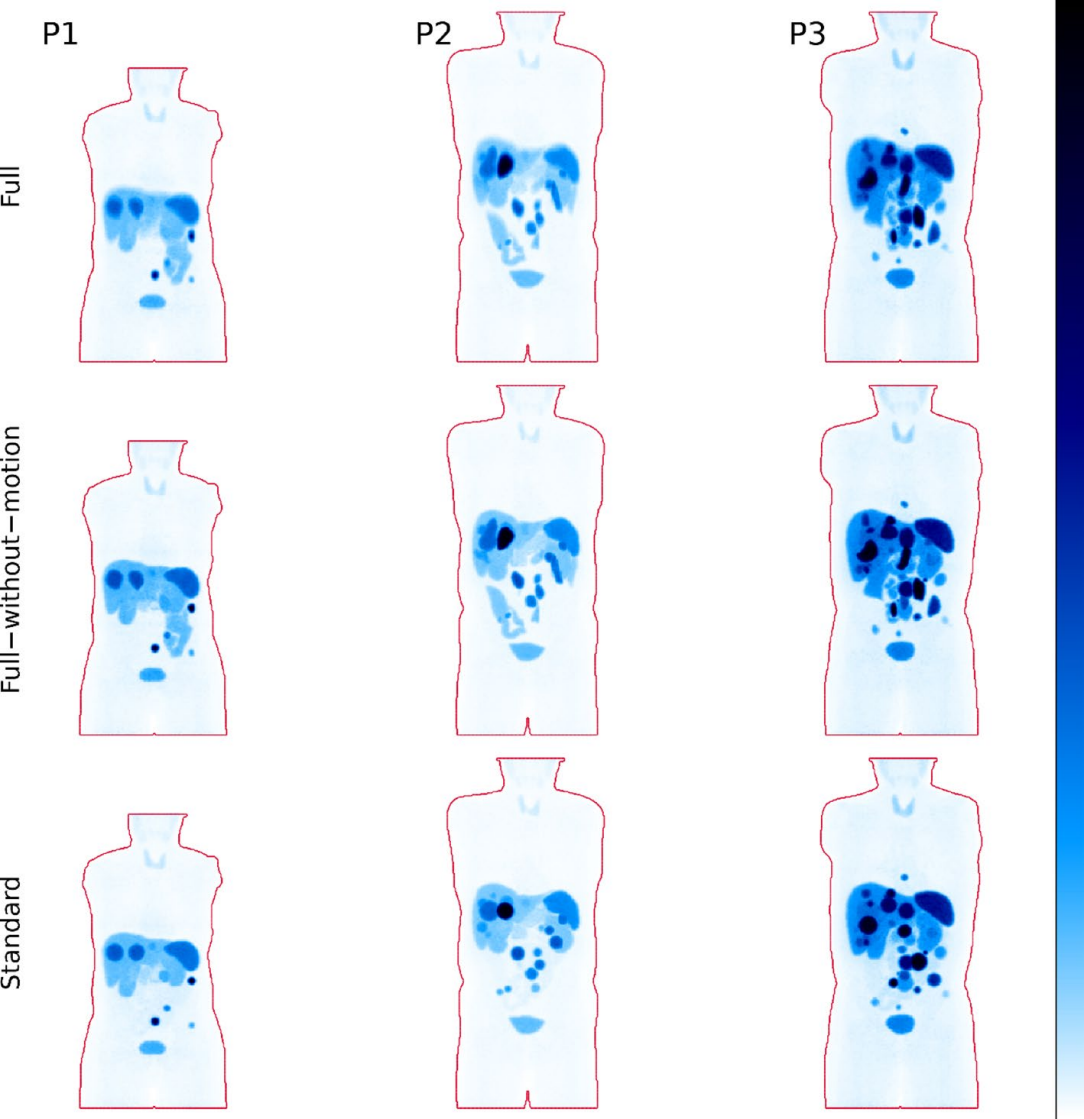
MIPs of simulated PET [^68^Ga]Ga-DOTA-TOC images for the three phantom models: Full (top row), full-without-motion (middle row), and standard (bottom row). Phantom contours are indicated in red. Colour scales are normalized within each column separately

**Fig. 7:**
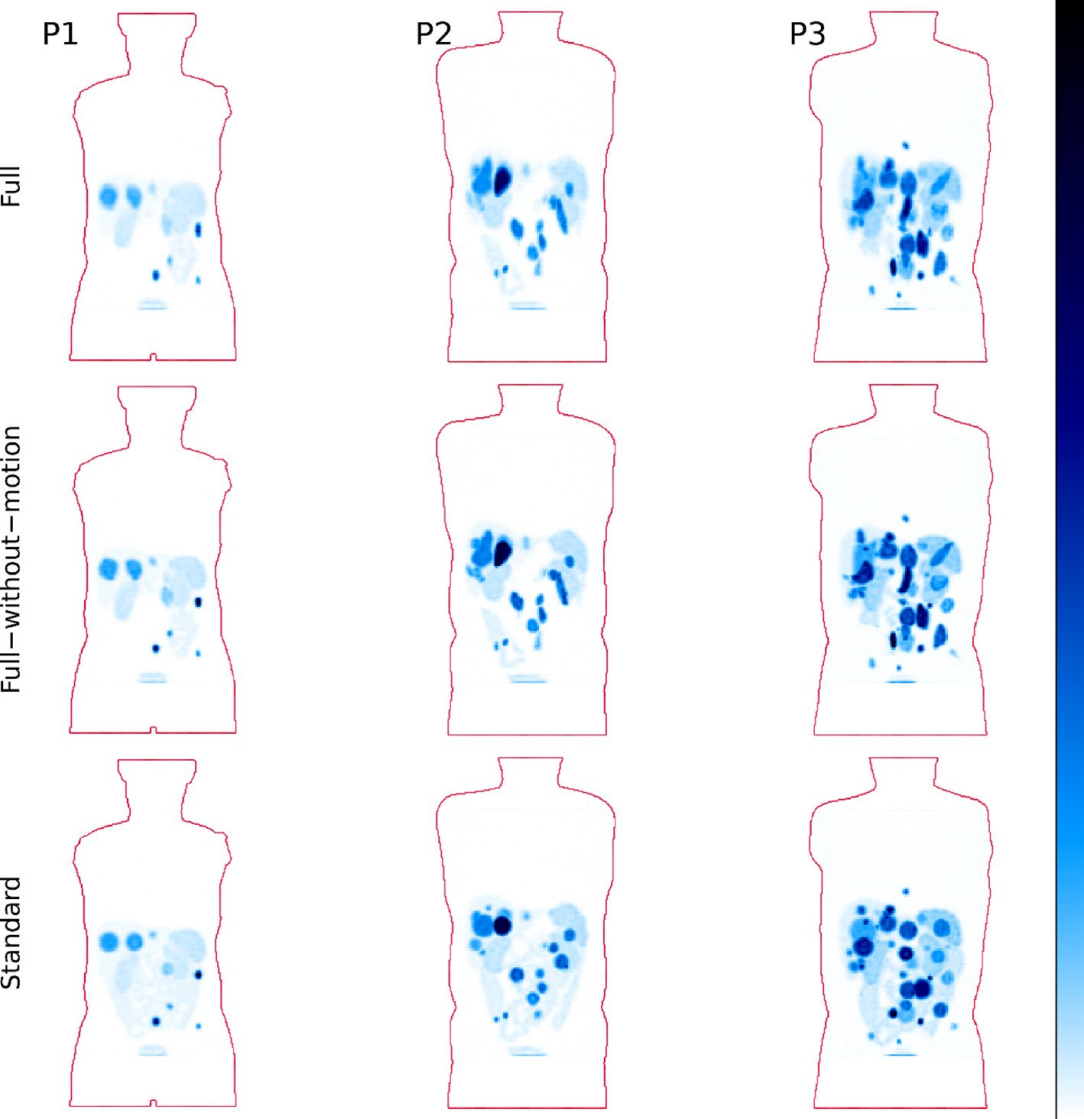
MIPs of simulated SPECT [^177^Lu]Lu-DOTA-TATE images for the three phantom models: Full (top row), full-without-motion (middle row), and standard (bottom row). Phantom contours are indicated in red. Colour scales are normalized within each column separately

Simulated ^68^Ga-SSTR PET images for the full models of P1, P2, and P3 are shown in Fig. 6, alongside a comparison with the phantoms without motion (full-without-motion and standard). The corresponding results for ^177^Lu-PRRT SPECT are shown in Fig. 7. By qualitative comparison of the full and full-without-motion models, the image intensity of tumours decreases when introducing respiratory motion, particularly for small tumours positioned in anatomic regions affected by motion-induced partial volume effects. Moreover, these tumours get an elongated appearance in images. Furthermore, the integration of realistic lesion shapes instead of spherical ones (standard) contribute to an image appearance that is substantially more similar to that encountered at patient imaging. In addition, the patchy uptake in the intestines of the refined models introduces variability and complexity that are non-existent in the standard phantom models.

The NEMA-phantom simulations and subsequent fitting according to Eq. 2 resulted in values of $\alpha$ and $\beta$ of 2.1 mL and 0.51 (PET) and 0.25 mL and 0.51 (SPECT).

### Errors in estimated lesion-activity concentration

**Fig. 8:**
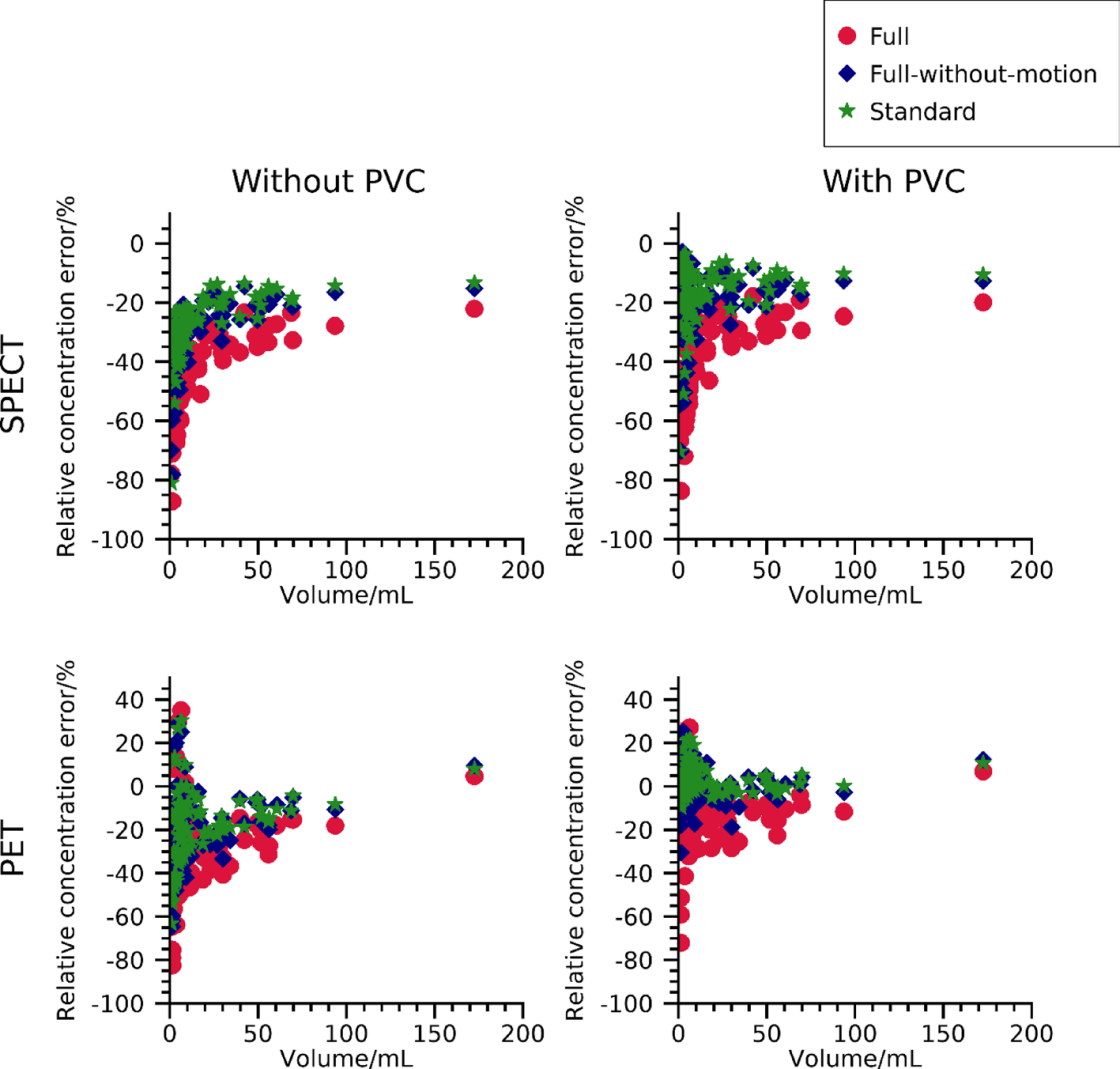
Errors in the activity concentration estimated from ^68^Ga-PET and ^177^Lu-SPECT images, as function of lesion volume for the three phantom models. Results are shown both with and without post-reconstruction PVC

Relative errors in the estimated activity concentration in lesions as function of volume are shown in Fig. 8. Results are presented for all three models for ^177^Lu-SPECT and ^68^Ga-PET images, with and without post-reconstruction PVC. Excluding PVC yields errors of between − 80% and − 15% for ^177^Lu-SPECT and between − 80% and + 40% for ^68^Ga-PET, with a pronounced volume dependence. When including PVC, errors obtained are in similar ranges but approach zero for the larger volumes for ^177^Lu-SPECT, whilst they are broadly centred around zero for ^68^Ga-PET. Comparing the effects of the three phantom models, the negative errors resulting from partial-volume effects are typically largest for the full model and smallest for the standard model. This suggests that respiratory motion has a major effect on activity-concentration errors, and that spherical lesions are insufficient to fully capture the errors that can be expected for patient studies. Table 4 shows the mean relative errors obtained for different volume ranges. For both ^68^Ga-PET and ^177^Lu-SPECT there are evident trends across volume intervals, where the full model yields the largest errors, the model full-without-motion yields lower errors, and the standard model yields the lowest errors. Again, this indicates that evaluation based on simplified phantom models may produce falsely low errors.

**Table 4:** Mean relative errors in the estimated lesion activity concentration. Results are separated into tumour-volume intervals of 0 mL to 10 mL, 10 mL to 20 mL, 20 mL to 50 mL, and larger than 50 mL

		0 mL–10 mL (43 tumours)	10 mL–20 mL (11 tumours)	20 mL–50 mL (13 tumours)	Larger than 50 mL (9 tumours)
^177^Lu-SPECT without PVC	Full	-51%	-40%	-33%	-29%
	Full-without-motion	-38%	-27%	-22%	-19%
	Standard	-34%	-25%	-20%	-17%
^177^Lu-SPECT with PVC	Full	-41%	-34%	-27%	-25%
	Full-without-motion	-25%	-19%	-16%	-15%
	Standard	-20%	-16%	-13%	-12%
^68^Ga-PET without PVC	Full	-34%	-12%	-29%	-19%
	Full-without-motion	-25%	-4%	-19%	-10%
	Standard	-23%	-3%	-16%	-9%
^68^Ga-PET with PVC	Full	-15%	-3%	-17%	-10%
	Full-without-motion	0%	8%	-3%	0%
	Standard	4%	9%	0%	1%

## Discussion

Monte Carlo simulations are immensely useful for the evaluation of quantitative PET and SPECT. Compared with physical phantom measurements, simulated images can imitate patient-like geometries that are difficult to achieve experimentally, although at the expense of the need for relying on computational models. Modern Monte Carlo-based simulation tools are well-validated and offer accurate models of the underlying physics for nuclear imaging [9, 40]. When combined with accurate models of camera equipment, good agreement with physical measurements is reached [41–43]. The limiting factor then becomes the extent to which the geometry and variation of patients can be mimicked.

The introduction of ^177^Lu-PRRT into clinical practice, along with its theragnostic association with ^68^Ga-SSTR imaging, has increased the interest in deriving quantitative and semi-quantitative metrics for therapy prediction, patient stratification, and patient-specific dosimetry [44–46]. Technical validation of the applied quantitative methods is essential, and Monte Carlo simulation here serves as an important tool. For such validation studies to hold merit, methods for designing sufficiently complex source geometries are needed. Whilst previous studies have improved the realism by considering specific steps in the phantom-generation process, the pipeline developed in this study aimed to combine several aspects, including respiratory motion, variability in tumour shape and size, and non-uniformity in activity concentrations. The impact of respiratory motion, non-spherical lesions and background non-uniformity can be appreciated when comparing simulated images in Figs. 6 and 7. Essentially, respiratory motion and tumour shape both increase the magnitude of partial-volume effects, associated with errors in the estimation of both volume and activity concentration [47–49].

**Fig. 9:**
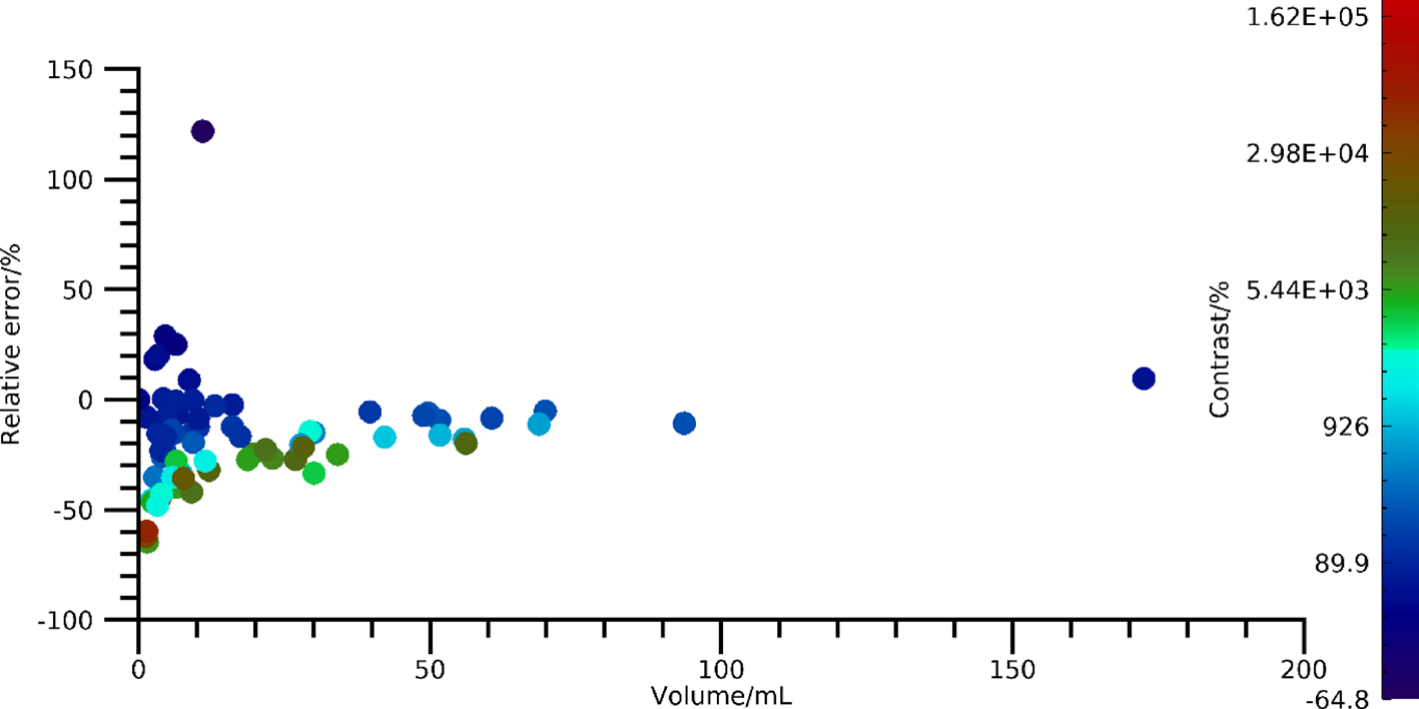
Relative errors for PET images simulated without motion, as function of volume and image contrast $\left( {C_{{{\mathrm{tum}}}} /C_{{{\mathrm{bkg}}}} - 1} \right)$, where $C_{{{\mathrm{tum}}}}$ is the tumour activity concentration and $C_{{{\mathrm{bkg}}}}$ is the mean concentration in the immediate background. The colour scale indicates image contrast

Estimated activity concentrations demonstrate a large range of errors for both PET and SPECT in Fig. 8. To some extent, this large range is caused by the size dependence of the partial-volume effect [39, 48], but some other effects are also noticeable. The importance of considering respiratory motion in image-based activity-concentration estimation is highlighted by Fig. 8 and Table 4, where estimates are consistently lower for this model than when motion is excluded. Effects of tumour shape are also visible in data, with spherical lesions demonstrating a lower bias than their non-spherical counterparts. Full method validation thus requires phantoms that account for both these effects. A further observation from Fig. 8 is that the relative error is positive for some lesions in PET data. This was found to be a result of spill-in from the background and the large range of contrasts present in the PET simulations. Figure 9 shows a more detailed analysis for ^68^Ga-PET images without motion, with errors as function of both tumour volume and contrast. As noted, positive errors mainly result for tumours with low or negative contrast, whilst tumours with a high contrast exhibit the familiar recovery curve that slowly converges to unity for increasing volume. This observation confirms the inclusion of image contrast in the parametrisation of partial-volume correction for ^68^Ga-PET images (Eq. 3), i.e., accounting for background and not only spill-out from the tumour. As noted in previous publications [27], the effect of background is much lower in the ^177^Lu-PRRT SPECT data. Table 4 demonstrates that the difference between the errors when including versus excluding PVC are larger for the full model than for the full-model-without-motion. In principle, this could indicate a sensitivity of the PVC process to uncertainty in the estimation of the background concentration, due to tumour motion. However, the difference in average background correction factor between the two phantom models was minor (15% versus 13% for PET, and 5% versus 3% for SPECT), and this is likely not the only cause of these differences in errors.

Two limitations in the developed patient models are that the nominal organ activity-concentration ratios are not varied between phantoms, and that the respiratory motion pattern is based on the same model for all phantoms. In principle, it would be possible to map the variability of the activity concentration in organs following the same procedure as applied for patient tumours. However, for SSTR-imaging, the organ activity concentration exhibits more pronounced differences between ^68^Ga-PET and ^177^Lu-SPECT than between patients, in particular for liver and kidneys, compared to tumors [50]. Thus, for other applications, the model will need to be revised for cases when between-patient organ variability is relevant. The primary motivation for the current study was to enable validation of methods for quantification of tumour data in ^68^Ga- and ^177^Lu-SSTR imaging, and using identical nominal ratios for organs was considered justified.

A problem when discussing tumour shapes and non-uniformities in activity concentration is the question of spatial scale. Whilst radiopharmaceuticals are known to distribute non-uniformly in tissues at a micro-scale [51, 52], the non-uniformities considered in the current study are on a macroscopic scale. The implemented non-uniformity models have no direct physiological basis and parameters were adjusted to mimic the character of patient images. Hence, there is no claim that the introduced non-uniformity has a physiological correspondence but rather describes a background variability observed in patient images. Since background variability in images is also affected by image acquisition and reconstruction settings [53], the model parameters may have to be tuned for application to other systems. Likewise, the lesions introduced in the phantoms were based on delineation in patient images using an active model based on Fourier descriptors [25]. Since the finite number of Fourier orders in the surface description intrinsically imposes a smoothness criterion on derived VOIs, the lesions will in practice also be at a limited spatial resolution in terms of shape and complexity.

Detailed computer phantoms are needed in applications relying on realistic image datasets with access to a well-defined ground, and the developed phantoms represent an important step forward for enabling simulation representative of patient images. Whilst the main application in this work is image-based activity quantification in ^68^Ga- and ^177^Lu-SSTR imaging, the developed pipeline is applicable for other applications as well, and offer support for the generation of training data for machine-learning applications [31, 54].

## Conclusions

A method for simulating images of XCAT-based patient models with respiratory motion, non-spherical tumour inserts, heterogenous inter-tumour activity concentration, and non-uniform intra-organ activity- concentration distributions has been developed. These developments improve the realism of simulated PET and SPECT images, which is important for, e.g., [^68^Ga]Ga-DOTA-TOC and [^177^Lu]Lu-DOTA-TATE image-based activity-concentration estimation. The most important factor is respiratory motion, while tumour shape and background non-uniformity have a minor impact for evaluation of activity concentration.

## Data Availability

Phantoms and images are available at www.msf.lu.se/en/research/resources.

## Abbreviations

FWHM: Full width at half maximum
MC: Monte Carlo
MIP: Maximum intensity projection
OS-EM: Ordered subsets expectation maximization
PET: Positron emission tomography
SPECT: Single photon emission computed tomography
PRRT: Peptide receptor radionuclide therapy
PVC: Partial-volume correction
SSTR: Somatostatin receptor
VOI: Volume-of-interest
